# Effects of Barefoot Walking on Menopausal Symptoms, Sleep Quality, Stress, and Quality of Life in Middle-Aged Women Experiencing Menopausal Symptoms

**DOI:** 10.3390/healthcare13222836

**Published:** 2025-11-08

**Authors:** Myoung-Hee Kim, Eun-Young Lee

**Affiliations:** Department of Nursing, Semyung University, 65 Semyung-ro, Jecheon-si 27136, Chungcheongbuk-do, Republic of Korea; mh1352@semyung.ac.kr

**Keywords:** menopause, sleep quality, stress, quality of life, barefoot walking

## Abstract

**Background**: The purpose of this study was to evaluate the effects of a structured barefoot walking program on menopausal symptoms, sleep quality, stress, and quality of life in middle-aged women experiencing menopausal symptoms. **Methods**: A quasi-experimental design with a nonequivalent control group and pretest-posttest was used. Community-dwelling women aged 45 to 65 years residing in Wonju, Republic of Korea, were recruited and assigned to either an experimental (*n* = 29) or control group (*n* = 31). The intervention consisted of 12 weeks of barefoot walking (three times per week, 60 min per session). Outcome measures included the Menopause Rating Scale (MRS), Verran and Snyder–Halpern Sleep Scale, Stress Response Inventory, and WHOQOL-BREF. In addition, changes in participants’ body composition before and after the intervention were assessed using a bioelectrical impedance analyzer (InBody 770). Data were analyzed using the Mann–Whitney U test, repeated measures ANOVA, ANCOVA, and other relevant statistical methods, with the level of significance set at *p* < 0.05. **Results**: Compared with the control group, the experimental group showed significant improvements in menopausal symptoms (z = −5.59, *p* < 0.001), stress (z = −3.58, *p* < 0.001), and quality of life (z = −3.47, *p* = 0.001). A significant time-by-group interaction effect was observed for sleep quality (F = 7.53, *p* = 0.008). No significant changes were found in body composition. **Conclusion**: Barefoot walking represents a promising, low-cost, community-based intervention for alleviating menopausal symptoms, enhancing sleep quality, reducing stress and improving quality of life in middle-aged women experiencing menopausal symptoms. Further randomized controlled trials are warranted to confirm these findings.

## 1. Introduction

Menopause is a natural biological transition in a woman’s life, typically occurring around the age of 50, and is associated with a variety of physical and psychological symptoms. With the average life expectancy of Korean women continuing to increase, improving the quality of life after menopause has emerged as a major public health concern [1].

According to the 2022 Women’s Health Survey conducted by the Korea Institute for Health and Social Affairs [2], a considerable proportion of Korean women in the menopausal transition reported experiencing menopausal symptoms. Using the Menopause Rating Scale (MRS), a symptom rated as “severe” or “very severe” (score of 3 or 4 on a 0–4 scale) was classified as a severe menopausal symptom. Among the 1307 peri- and postmenopausal respondents, 28.0% reported severe sleep problems, 27.2% reported vaginal dryness, and 23.3% experienced joint or muscle discomfort, while notable proportions also reported fatigue (21.5%) and hot flashes (19.7%).

Despite the severity, only 19.5% of these women had sought medical treatment for their symptoms. In an effort to alleviate their menopausal symptoms, many women reported using dietary supplements or engaging in physical exercise. However, a substantial proportion (34.3%) indicated that they did not employ any specific method to manage their symptoms. These findings highlight a gap in awareness or accessibility of effective menopausal health management strategies among midlife Korean women. Furthermore, postmenopausal women are at a significantly higher risk of developing chronic conditions such as osteoporosis, cardiovascular diseases, insomnia, and depression, indicating the necessity for proactive health management strategies [1,3].

Recent evidence indicates that a large proportion of menopausal and postmenopausal women experience insomnia, anxiety, and depressive symptoms, which together impair daily functioning and reduce quality of life while increasing long-term health risks. Meta-analytic and systematic reviews have shown that sleep disturbances are highly prevalent during the menopausal transition and often persist, contributing to daytime fatigue, cognitive complaints, mood disorders, and cardiometabolic risk [4,5].

Psychological distress—including elevated stress, anxiety, and depressive symptoms—has been shown to both exacerbate vasomotor and sleep-related complaints and mediate the relationship between hot flashes and poor sleep, suggesting an important psychophysiological pathway linking menopausal symptoms to reduced well-being [6,7]. Consequently, menopausal symptoms encompass not only physiological discomfort but also a substantial psychological burden that can diminish overall quality of life and increase feelings of social isolation.

Randomized controlled trials and meta-analyses further indicate that lifestyle, educational, and exercise interventions can alleviate menopausal symptoms and improve sleep and quality of life, underscoring the need for integrated, evidence-based programs that address both the physical and psychological dimensions of menopause, particularly in community settings [8,9].

Menopausal symptoms are not limited to physiological discomfort but may also result in increased stress levels, diminished quality of life, and feelings of social isolation [6]. Therefore, an integrated approach that addresses both the physical and psychological aspects of menopause is crucial. Evidence-based interventions specifically designed to enhance the health and well-being of menopausal women are urgently needed, along with practical, applicable programs that can be implemented in community settings.

Recent non-surgical interventions for alleviating menopausal symptoms and improving quality of life have garnered increasing attention. Structured exercise, yoga, cognitive behavioral therapy, and lifestyle modifications consistently demonstrate beneficial effects on sleep, emotional states, autonomic regulation, and overall symptom burden in menopausal women [10,11,12]. For example, a meta-analysis examining the effects of exercise on sleep in menopausal women found that physical activity significantly improved sleep quality and reduced insomnia symptoms [13]. In addition, other studies have reported that exercise interventions generally decrease the severity of menopausal symptoms, including mood disturbances and vasomotor complaints [14].

In parallel with these approaches, “grounding,” or barefoot contact with the earth, has been investigated in preliminary studies as a low-risk adjunctive strategy to modulate stress physiology, normalize cortisol rhythms, and enhance sleep quality. One of the earliest studies by Ghaly and Teplitz [15] reported that 8 weeks of grounding during sleep lowered nighttime cortisol levels and re-synchronized diurnal cortisol patterns in a small sample, accompanied by improvements in self-reported sleep, pain, and stress. More recent experimental and review evidence suggests that grounding or earthing may shift autonomic balance toward parasympathetic dominance, reduce inflammatory markers, and regulate cortisol [16]. For instance, Chevalier et al. [16] demonstrated reductions in pain and alterations in immune cell profiles associated with grounding interventions.

The proposed mechanisms underlying the effects of grounding involve direct physical contact between the human body and the earth’s surface electrons, which may facilitate the neutralization of positively charged free radicals and restore the body’s electrical equilibrium [16,17]. This process has been hypothesized to attenuate oxidative stress and inflammatory responses, thereby lowering sympathetic nervous system activity and cortisol secretion. In addition, skin contact with the ground may influence electrophysiological properties—such as reduced muscle tension and improved vagal tone—leading to parasympathetic activation and improved stress regulation. Through these pathways, barefoot contact with natural surfaces may contribute to reduced physiological arousal and enhanced endocrine stability, offering a plausible mechanism for the observed reductions in stress and normalization of cortisol levels.

However, these grounding studies predominantly target healthy adults or other specific populations rather than menopausal women [16,18]. Therefore, the present study aims to examine whether a barefoot walking (grounding) intervention can effectively reduce menopausal symptoms, improve sleep quality, alleviate stress, and enhance quality of life in menopausal women. Such an investigation could position barefoot walking as a cost-effective, scalable, community-based health promotion strategy and clarify its complementary role alongside established non-pharmacological interventions.

Accordingly, the present study aimed to investigate the effects of a 12-week barefoot walking intervention on menopausal symptoms, sleep quality, stress, and quality of life among middle-aged women experiencing menopausal symptoms. The primary objective was to examine whether barefoot walking would improve overall menopausal symptoms—specifically vasomotor (e.g., hot flashes, sweating), psychological (e.g., irritability, anxiety, depressed mood), and somatic (e.g., joint and muscle discomfort, sleep disturbance) domains—operationally defined and assessed using the Menopause Rating Scale (MRS). A total score of 8 or higher on the MRS was considered indicative of the presence of menopausal symptoms, with higher scores reflecting greater severity.

The secondary objectives were to evaluate whether barefoot walking, compared with no intervention, would improve sleep quality as measured by the Verran and Snyder–Halpern (VSH) Sleep Scale, reduce perceived stress as assessed by the Stress Response Inventory (SRI), and enhance quality of life as measured by the World Health Organization Quality of Life Scale—Abbreviated Version (WHOQOL-BREF).

Based on these objectives, the following hypotheses were tested: (1) Participants in the barefoot walking group will demonstrate a greater reduction in menopausal symptoms compared to those in the control group. (2) Participants in the barefoot walking group will exhibit improved sleep quality relative to the control group. (3) Participants in the barefoot walking group will show lower levels of perceived stress compared to the control group. (4) Participants in the barefoot walking group will report a higher quality of life than those in the control group.

## 2. Materials and Methods

### 2.1. Study Design

This study employed a quasi-experimental design using a nonequivalent control group with a pretest-posttest structure to examine the effects of barefoot walking on menopausal symptoms, sleep quality, stress, and quality of life among middle-aged women experiencing menopausal symptoms.

### 2.2. Setting and Sample

This study targeted community-dwelling middle-aged women aged 45 to 65 years residing in Wonju, Republic of Korea, who were experiencing menopausal symptoms. The inclusion criteria were as follows: (1) women aged between 45 and 65 years; (2) women who scored ≥8 on the Korean version of the Menopause Rating Scale, indicating the presence of menopausal symptoms; and (3) individuals able to participate in walking exercise and provide written informed consent. Participants were not restricted to those who had reached complete menopause (defined as the absence of menstruation for 12 consecutive months). Eligibility was determined based on the presence of menopausal symptoms, regardless of menstrual status, as assessed using the MRS. Thus, both perimenopausal and postmenopausal women were included if they met the symptom severity criterion (MRS ≥ 8) and other inclusion requirements. This approach was adopted because the study focused on the experience and management of menopausal symptoms rather than the physiological menopausal status.

The exclusion criteria were as follows: (1) having medical conditions that limited physical activity; (2) currently receiving hormone replacement therapy or psychiatric treatment; (3) engaging in any form of structured or habitual physical exercise, such as aerobic exercise, fitness training, or walking, at least three times per week, based on self-reported activity patterns obtained during the screening interview; and (4) having cognitive impairments that could interfere with participation or the completion of questionnaires. This exclusion criterion regarding regular exercise was intended to ensure that participants were not already habitually engaged in other consistent exercise routines, as the purpose of the study was to examine the independent effects of a newly introduced barefoot walking program.

Participants were recruited from various community institutions within Wonju, Republic of Korea, through cooperation with local organizations to ensure feasibility in a community-based setting.

The experimental group consisted of women who participated in barefoot walking programs implemented in two phases. The first program, for community-recruited participants, was conducted over a 12-week period beginning in June 2023. The second program, for employees of the Health Insurance Review & Assessment Service (HIRA), was conducted over 12 weeks beginning in April 2024.

The control group participants were recruited from HIRA, the Rehabilitation and Welfare Center for People with Disabilities, and a community health center. Data collection for control participants at HIRA and the Rehabilitation and Welfare Center began in November 2023, while data collection at the community health center commenced in February 2024. Control group participants were instructed to maintain their usual daily routines and to refrain from engaging in barefoot walking or any new structured exercise programs during the study period.

At the HIRA institution, data were collected sequentially to prevent contamination between groups. After completing data collection for the control group, the research team initiated a new recruitment phase for the barefoot walking program. Some participants who had previously taken part in the control group expressed interest in the intervention and were re-enrolled as members of the experimental group after the control phase ended. Their subsequent 12-week barefoot walking data were analyzed as part of the experimental group dataset.

Although the data collection periods for the experimental and control groups were not entirely concurrent, both groups were recruited from the same community population within overlapping timeframes. The same inclusion and exclusion criteria were applied to ensure comparability and to minimize potential selection bias in this community-based quasi-experimental design.

To determine the appropriate sample size for this study, an a priori power analysis was conducted using G*Power 3.1 [19]. The statistical test selected was repeated measures ANOVA, considering a two-group (intervention vs. control) pretest-posttest design. Based on Cohen’s guidelines, a medium effect size (f = 0.25), a significance level of α = 0.05, and a statistical power of 0.95 were set [20]. The number of measurements was set to two (pre- and post-intervention), and the correlation among repeated measures was assumed to be 0.5. The nonsphericity correction epsilon (ε) was set to 1, assuming sphericity. The results of the power analysis indicated that a minimum total sample size of 54 participants (*n* = 27 per group) was required to achieve a statistical power of 0.95 (effect size f = 0.25, α = 0.05). Considering an anticipated dropout rate of 30% in the experimental group and 20% in the control group, a total of 73 participants were enrolled in the study after screening for eligibility. Among the 91 individuals initially assessed, 18 were excluded for not meeting the inclusion criteria (Menopause Rating Scale [MRS] score < 8, *n* = 16) or declining to participate (*n* = 2). In total, 39 women were assigned to the experimental (barefoot walking) group, comprising 25 community participants and 14 employees from the Health Insurance Review & Assessment Service. In total, 34 women were assigned to the control group, including 5 participants from the Health Insurance Review & Assessment Service, 19 from the Rehabilitation and Welfare Center for People with Disabilities, and 10 from a community health center. During follow-up, 10 participants in the experimental group discontinued participation before completing the 12-week barefoot walking program, and 3 participants in the control group did not complete the post-test assessment conducted 12 weeks after the baseline survey. Consequently, the final analysis included 29 participants in the experimental group and 31 in the control group.

### 2.3. Ethical Consideration

This study was approved by the Institutional Review Board (IRB) of Semyung University (IRB No. SMU-2023-03-003-01, approval date: 7 April 2023) and conducted in accordance with the ethical principles outlined in the Declaration of Helsinki. All participants were informed about the study’s purpose, procedures, potential risks and benefits, and their right to withdraw at any time. Written informed consent was obtained from all participants prior to enrollment. This study was conducted as a community-based quasi-experimental intervention rather than a clinical trial. Therefore, it was not preregistered in a clinical trial registry. The absence of preregistration may increase the risk of outcome reporting bias; however, all study procedures, measurements, and analyses were conducted according to the protocol approved by the Institutional Review Board of Semyung University (IRB No. SMU-2023-03-003-01).

### 2.4. Barefoot Walking Intervention

The barefoot walking program was developed based on previous studies [21,22,23,24,25,26,27], and was implemented three times per week for 60 min per session over a 12-week period. In accordance with the guidelines of the American College of Sports Medicine for exercise testing and prescription [28], the program was structured to include three core components: warm-up, main exercise, and cool-down. The warm-up consisted of light stretching, the main exercise involved barefoot walking, and the cool-down included breathing, relaxation, and foot washing (Table 1).

The barefoot walking program was developed in consultation with a certified barefoot walking instructor affiliated with the Korea Walking & Trail Association. The program emphasized the benefits of barefoot walking derived from prolonged contact with the ground. To minimize impact and prevent potential injuries, participants were instructed to walk at a slower pace than their usual walking speed and to maintain a conversational pace without experiencing significant fatigue or pain [27].

The intervention was conducted at the Ungok Solbaram Forest Trail in Wonju City, a 2.7 km forest path designed and maintained by the municipality specifically for barefoot walking. The forest trail consists of well-maintained soil paths specifically designed for barefoot walking, providing a natural and moderately soft texture that facilitates safe grounding and sensory stimulation. The route is shaded by pine trees and equipped with foot-washing facilities located at the entrance, which also serves as the exit, providing a suitable environment for barefoot walking. The average walking time to complete one loop was approximately 50 min, corresponding to a slow to moderate walking intensity. At study enrollment, participants were provided with a YouTube instructional video demonstrating proper barefoot walking techniques (https://www.youtube.com/watch?v=tcfebXsgNSA&t=213s) (accessed on 2 June 2023).

During the first session, participants practiced under the direct supervision of the research assistant, who was also a certified barefoot walking instructor, and subsequently continued independent walking sessions while maintaining a daily walking log. The research assistant monitored participants’ adherence and safety through an SNS group chat (Band) and provided weekly reminders and feedback. During the study period, there were no reports of injuries, discomfort, or adverse events among participants. The research assistant, who managed the study participants, holds an official Walking Instructor Certificate issued by the Korea Walking & Trail Association and has over five years of experience conducting community-based walking and barefoot walking programs.

### 2.5. Study Procedures

The implementation of the barefoot walking program and data collection were carried out by a certified barefoot walking instructor who served as a research assistant for this study.

#### 2.5.1. Barefoot Walking Group

The barefoot walking program for community-recruited participants was conducted over a 12-week period beginning in June 2023, while the program for participants from the Health Insurance Review & Assessment Service was implemented for 12 weeks starting in April 2024. To facilitate communication and monitor participation, all enrolled participants were managed through a group messaging platform (Band, a social networking service).

As part of participant safety measures and compensation, individuals in the experimental group were provided with support for tetanus vaccination prior to the start of the intervention. Although barefoot walking was conducted safely on a well-maintained forest trail, participants were barefoot during the sessions, and there was a potential, albeit minimal, risk of minor skin injuries that could serve as an entry route for Clostridium tetani. The tetanus vaccination was therefore provided as a precautionary measure to prevent infection. In recent years, as barefoot walking has gained popularity in Korea, several local public health centers and health authorities have recommended tetanus vaccination prior to participating in barefoot walking activities as part of their community safety guidelines. To ensure correct barefoot walking practices, a video demonstrating proper techniques—produced by the researcher in collaboration with the certified barefoot walking instructor who served as a research assistant—was provided to all participants for viewing before the program began.

Pre-intervention data were collected prior to the start of the barefoot walking program. Participants completed a structured questionnaire assessing general characteristics, menopausal symptoms, sleep quality, stress levels, and quality of life. Body composition was measured at the local public health center under the guidance of the research assistant using a bioelectrical impedance analyzer (InBody 770, Biospace Co., Seoul, Republic of Korea). Participants were instructed to submit their printed measurement results to the research assistant immediately after the assessment.

Throughout the 12-week intervention period, the researcher and the research assistant (a certified barefoot walking instructor) encouraged participants to maintain barefoot walking diaries to monitor adherence and promote continued engagement. In addition, support and communication were provided via the Band social networking service, where participants could ask questions and receive feedback related to barefoot walking. To minimize the influence of environmental factors, participants were instructed to refrain from engaging in other forms of physical exercise during the intervention period.

Post-intervention assessments were conducted immediately after the completion of the 12-week program. Participants again completed the same questionnaires on menopausal symptoms, sleep quality, stress, and quality of life. Body composition was remeasured at the same health center following the same standardized procedure as at baseline.

#### 2.5.2. Control Group

Data collection for participants from the Health Insurance Review & Assessment Service and the Rehabilitation and Welfare Center for People with Disabilities began in November 2023, while data collection at the community health center commenced in February 2024.

Participants who provided informed consent completed a baseline assessment that included measures of general characteristics, menopausal symptoms, sleep quality, stress levels, and quality of life. Body composition was assessed at the public health center using the same bioelectrical impedance analyzer (InBody 770, Biospace Co., Seoul, Republic of Korea) under the supervision of the research assistant, and participants were instructed to provide their measurement reports to the research assistant.

To minimize the influence of environmental factors, participants in the control group were instructed to refrain from engaging in any intentional or structured physical activity beyond their usual daily routines during the study period.

A follow-up assessment was conducted 12 weeks after the baseline assessment. Participants completed the same questionnaires and underwent the same body composition measurement procedures as at baseline. As a form of compensation, participants in the control group received a transportation allowance of 10,000 KRW upon completion of the post-assessment.

### 2.6. Measurements/Instruments

In the baseline questionnaire, participants’ general characteristics—including age, height, weight, religion, educational level, employment status, and marital status—were collected. These data were used to describe the sample and to examine the homogeneity between the experimental and control groups.

#### 2.6.1. Menopausal Symptoms

Menopausal symptoms were assessed using the Korean version of the Menopause Rating Scale (MRS), developed by Heinemann et al. [29]. The MRS is a self-reported screening tool designed to evaluate menopausal symptoms and consists of three subscales: somato-vegetative (4 items), urogenital (3 items), and psychological (4 items), totaling 11 items. Each item is rated on a 5-point Likert scale ranging from 0 (no symptoms) to 4 (very severe), with a total possible score ranging from 0 to 44. Higher scores indicate more severe perceived menopausal symptoms. Total scores are interpreted as follows: 0–4 (no or minimal symptoms), 5–7 (mild), 8–15 (moderate), and ≥16 (severe). The internal consistency reliability (Cronbach’s α) was 0.86 for the original instrument and 0.85 in the present study.

#### 2.6.2. Sleep Quality

The quality of sleep was assessed using the Verran and Snyder–Halpern (VSH) Sleep Scale, originally developed by Snyder–Halpern and Verran [30]. The instrument consists of 8 items rated on a 10-point numeric rating scale, where higher scores indicate better subjective sleep quality. The items assess subjective experiences related to sleep during the night, including “no awakening during sleep,” “no movement during sleep,” “total hours of sleep,” “depth of sleep,” “falling asleep immediately upon lying down,” “waking up feeling refreshed,” “waking up spontaneously,” and “satisfaction with sleep.”

At the time of its development, the internal consistency reliability of the instrument was Cronbach’s α = 0.82. In the present study, the Cronbach’s α was 0.85.

#### 2.6.3. Stress

Stress levels were measured using the Stress Response Inventory (SRI) developed by Koh et al. [31]. This instrument assesses stress responses experienced over the past week and consists of 39 items covering seven subscales: tension, aggression, somatization, anger, depression, fatigue, and frustration. Each item is rated on a 5-point Likert scale ranging from 0 (“not at all”) to 4 (“absolutely”), with total scores ranging from 0 to 156. Higher scores indicate greater levels of stress response. At the time of its development, the internal consistency reliability of the instrument was Cronbach’s α = 0.97. In the present study, the Cronbach’s α was 0.98.

#### 2.6.4. Quality of Life

The quality of life was measured using the Korean version of the WHO Quality of Life Scale Abbreviated Version (WHOQOL-BREF), which was originally developed by The WHOQOL Group [32] and standardized into Korean by Min et al. [33]. This instrument consists of 24 items across four domains: physical health (7 items), psychological health (6 items), social relationships (3 items), and environmental factors (8 items). Each item is rated on a 5-point Likert scale, with total scores ranging from 24 to 120, where higher scores indicate a better quality of life. The internal consistency reliability (Cronbach’s α) reported by Min et al. [33] was 0.89. In the present study, the Cronbach’s α was 0.94.

### 2.7. Data Analysis

All statistical analyses were performed using SPSS for Windows, Version 25.0 (IBM Corp., Armonk, NY, USA). The normality of all variables was assessed using the Shapiro–Wilk test. Between-group homogeneity at baseline was examined using the chi-square test, Fisher’s exact test, independent t-test, or Mann–Whitney U test, as appropriate.

To evaluate the effects of barefoot walking, analyses were organized around the primary and secondary outcomes. The primary outcomes included menopausal symptoms, sleep quality, stress, and quality of life, while the secondary outcomes were the body composition indices measured using bioelectrical impedance analysis.

For the primary outcomes, between-group comparisons of changes from baseline to post-intervention (Δ = post − pre) were conducted. The Mann–Whitney U test was applied to non-normally distributed variables (menopausal symptoms, stress, and quality of life), and repeated-measures ANOVA was used for normally distributed variables (sleep quality). For the secondary outcomes, repeated-measures ANOVA was employed for variables that met both normality and homogeneity assumptions (BMI, SMM, FFM, TBW, and BMR), whereas ANCOVA was applied when baseline differences were present (BFM), with baseline scores entered as covariates.

Effect sizes (r for Mann–Whitney U tests and partial η^2^ for ANOVA/ANCOVA) and their 95% confidence intervals were reported to indicate the magnitude and precision of the effects. For non-parametric tests, 95% confidence intervals for r were estimated using Fisher’s z-transformation approximation based on the total sample size.

All analyses were conducted on a per-protocol basis, including participants who completed both pre- and post-intervention assessments. The internal consistency reliability of the instruments was verified using Cronbach’s α.

## 3. Results

### 3.1. General Characteristics and Baseline Homogeneity Test of the Study Groups

The mean age of participants was 56.86 years (SD = 5.18) in the experimental group and 56.26 years (SD = 4.50) in the control group. Results of the homogeneity tests on general characteristics showed no statistically significant differences between the groups in age, height, weight, religion, educational level, employment status, or marital status, indicating that the two groups were homogeneous.

At baseline, there were no significant differences between the experimental and control groups in the primary outcome variables of this study: menopausal symptoms, sleep quality, stress, and quality of life.

Regarding the secondary outcome variables (body composition indices), there were no significant differences between the groups in body mass index (BMI), skeletal muscle mass (SMM), fat-free mass (FFM), total body water (TBW), or basal metabolic rate (BMR). However, a significant difference was found in body fat mass (BFM) between the groups at baseline (Table 2).

### 3.2. Effects of Barefoot Walking on Primary Outcomes

To assess the effects of barefoot walking on the primary outcomes—menopausal symptoms, sleep quality, stress, and quality of life—nonparametric and parametric analyses were conducted according to data distribution.

#### 3.2.1. Menopausal Symptoms, Stress, and Quality of Life

The Mann–Whitney U test was performed on change scores (Δ = post − pre) to compare the experimental and control groups (Table 3). The MRS scores significantly decreased in the experimental group, with a median reduction of 11.0 (IQR = 7.50), compared to a median reduction of 0.0 (IQR = 6.00) in the control group (U = 73.00, z = −5.59, *p* < 0.001, r = −0.72).

Similarly, stress levels significantly decreased in the experimental group, with a median reduction of 24.0 (IQR = 54.50), compared to 0.0 (IQR = 15.25) in the control group (U = 207.50, z = −3.58, *p* < 0.001, r = −0.46). Quality of life significantly improved in the experimental group, with a median increase of 15.0 (IQR = 25.00), compared to 2.5 (IQR = 10.75) in the control group (U = 215.50, z = −3.47, *p* = 0.001, r = −0.45).

These findings indicate that barefoot walking effectively alleviates menopausal symptoms, reduces stress, and enhances quality of life among middle-aged women experiencing menopausal symptoms.

#### 3.2.2. Sleep Quality

A repeated-measures ANOVA was conducted to examine the effect of barefoot walking on sleep quality (Table 4). The main effect of group was not significant (F = 0.94, *p* = 0.337, partial η^2^ = 0.016). However, a significant interaction between time and group was observed (F = 7.53, *p* = 0.008, partial η^2^ = 0.115), indicating that the experimental group demonstrated a greater improvement in sleep quality compared to the control group. These results support the hypothesis that barefoot walking positively influences sleep quality in middle-aged women.

### 3.3. Effects of Barefoot Walking on Secondary Outcomes (Body Composition)

The secondary outcomes, consisting of body composition indices measured using bioelectrical impedance analysis, were analyzed to determine the effects of barefoot walking on participants’ physical parameters (Table 5).

Repeated-measures ANOVA was used to evaluate BMI, SMM, FFM, TBW, and BMR. The analyses revealed no significant time × group interactions for any of these variables, indicating that barefoot walking did not produce significant changes in these body composition indices.

Because the assumption of baseline homogeneity was not met for BFM, ANCOVA was performed using pretest scores as covariates. Levene’s test confirmed that the assumption of homogeneity of variances was satisfied (*p* > 0.05). However, after adjusting for baseline differences, no statistically significant between-group difference was observed in BFM (F = 1.01, *p* = 0.321, partial η^2^ = 0.018). These findings suggest that the 12-week barefoot walking program did not significantly affect body composition among middle-aged women.

## 4. Discussion

This 12-week barefoot walking program produced clinically meaningful improvements in the primary outcomes among middle-aged women experiencing menopausal symptoms. Compared with the control group, the intervention group showed larger reductions in menopausal symptoms and stress and greater gains in quality of life, with a significant Group × Time interaction for sleep quality indicating greater improvement over time in the intervention group. By contrast, secondary outcomes (body composition indices) showed no significant Group × Time effects, and the ANCOVA for body fat mass, adjusting for baseline differences, was non-significant.

Overall, these findings indicate that a structured barefoot walking program was effective in improving menopausal symptoms, sleep quality, stress, and quality of life compared with no structured activity among middle-aged women experiencing menopausal symptoms. Participants in the barefoot walking group showed significant reductions in menopausal symptom severity and stress levels, along with meaningful improvements in sleep quality and overall well-being relative to the control group. However, no significant changes were observed in the secondary outcome of body composition over the 12-week intervention period, suggesting that barefoot walking primarily provides psychosomatic rather than body compositional benefits in this population.

Our results are consistent with prior evidence that exercise alleviates menopausal symptoms and improves sleep-related outcomes. Recent systematic reviews and meta-analyses have demonstrated that aerobic and mind–body exercise interventions significantly reduce insomnia severity and improve overall sleep quality in peri- and postmenopausal women [10,11,34,35]. Similarly, structured physical activity programs have been associated with reductions in vasomotor and psychological symptoms and with improvements in quality of life across the climacteric transition [36,37]. The existing literature suggests that regular exercise may serve as an effective nonpharmacological approach for alleviating menopausal symptoms, improving sleep, managing stress, and enhancing overall quality of life.

However, not all walking interventions in midlife women yield uniformly positive results; outcomes often vary depending on baseline symptom severity, adherence, and program dose (e.g., frequency, duration, and intensity). For example, Wilbur et al. [38] conducted a 24-week moderate-intensity walking trial among sedentary midlife women and found no overall group differences in menopausal symptom change, although greater adherence and higher baseline symptom severity predicted greater improvements in sleep. Although prior studies have reported mixed findings, the present study adds to the existing literature by providing evidence that barefoot walking may be an effective approach for managing menopausal symptoms in middle-aged women.

Regarding body composition, our null findings over 12 weeks accord with evidence that aerobic training alone at modest doses is often insufficient to alter fat or lean mass in midlife women without higher-volume resistance training or concurrent dietary modification [39,40].

To date, few studies have examined barefoot walking as an intervention for menopausal symptoms, sleep, stress, or quality of life in peri- or postmenopausal women. Existing evidence remains preliminary but promising. A quasi-experimental study conducted in urban forests demonstrated that barefoot walking significantly improved stress responses, mood states, sleep satisfaction, and quality of life compared with shod walking, suggesting additive psychological and physiological benefits derived from direct foot-ground contact and natural environmental exposure [41]. Complementing these findings, a qualitative study involving middle-aged women experiencing menopause reported that barefoot walking contributed to relief from chronic pain, better sleep quality, and enhanced overall well-being, highlighting its potential as a natural and restorative form of exercise [42]. Although randomized controlled trials remain lacking, these studies collectively indicate that barefoot walking may serve as a safe, accessible, and potentially effective nonpharmacological approach for alleviating menopausal symptoms, improving sleep, reducing stress, and enhancing quality of life among midlife women.

A growing body of grounding or earthing research suggests several complementary physiological mechanisms that may explain the beneficial effects observed in this study. Direct skin contact with the Earth’s surface allows the transfer of free electrons, which may act as antioxidants, neutralizing reactive oxygen species and attenuating chronic inflammation. This “universal anti-inflammatory” effect has been proposed as a central mechanism for improved pain regulation, autonomic stability, and emotional well-being [16,17,43,44]. Such antioxidative and anti-inflammatory actions could contribute to the reductions in menopausal symptoms and stress, and to the enhancement of overall quality of life observed among participants.

Grounding has also been reported to influence neuroendocrine and autonomic function, shifting balance toward parasympathetic dominance and normalizing diurnal cortisol patterns, both of which are closely linked to sleep regulation and stress resilience [16,43]. Furthermore, several studies have documented improved blood rheology and microcirculatory flow following grounding, which may support thermoregulation and tissue oxygenation—potentially mitigating vasomotor symptoms and fatigue [16,43]. Additional evidence suggests that grounding can modulate immune responses, reducing leukocytosis and inflammatory warmth after tissue injury, thereby supporting recovery and homeostasis [17,44]. Collectively, these findings provide a plausible physiological rationale linking barefoot walking—a natural form of grounding—with reduced menopausal symptoms, improved sleep, decreased stress, and enhanced quality of life in midlife women [16,17,43,44].

However, it should be noted that the proposed mechanisms remain largely theoretical, and much of the current evidence derives from small-scale studies, observational data, or reports involving potential conflicts of interest [17,43]. Therefore, the present discussion interprets these pathways as “possible physiological mechanisms” rather than confirmed causal explanations. Future randomized controlled trials incorporating biological markers—such as inflammatory cytokines, cortisol profiles, heart rate variability, or blood viscosity—are warranted to empirically validate these hypotheses and to strengthen the mechanistic understanding of barefoot walking interventions in menopausal women.

From a clinical and public health perspective, the findings of this study suggest that barefoot walking can be considered a simple, low-cost, and scalable nonpharmacological intervention for midlife women experiencing menopausal symptoms. For women who cannot or prefer not to use hormone therapy, regular barefoot walking offers a feasible alternative that promotes both physical and psychological well-being. As our data indicate, meaningful improvements in menopausal symptom severity, sleep quality, and stress occurred without adverse events or the need for specialized equipment, highlighting the practicality of incorporating barefoot walking into lifestyle-based menopause management programs.

Given that the program was conducted in a natural outdoor setting, barefoot walking may also yield synergistic benefits through combined exposure to nature, moderate physical activity, and grounding effects, which together foster relaxation, emotional balance, and social engagement. Such holistic engagement aligns with lifestyle medicine approaches emphasizing movement, stress reduction, and reconnection with natural environments [43,44]. Clinicians, nurses, and community health educators can therefore integrate barefoot walking sessions into community-based health promotion programs, particularly targeting peri- and postmenopausal women seeking nonpharmacological symptom management strategies.

Moreover, the absence of significant changes in body composition underscores that barefoot walking should be primarily recommended for its psychosomatic and quality-of-life benefits rather than for body fat reduction. Nonetheless, its accessibility and safety make it an attractive entry point for sedentary midlife women to begin regular activity, potentially enhancing long-term adherence to healthy movement patterns. Implementing structured, supervised barefoot walking programs in community settings—such as forest trails, parks, or wellness centers—could serve as a practical, evidence-based approach to promoting menopausal health and psychological resilience in this population.

Nevertheless, several limitations should be acknowledged. The quasi-experimental design precludes definitive causal inference and introduces potential selection bias due to nonrandom allocation. Although baseline homogeneity was verified, unmeasured confounding factors—such as dietary habits, stress exposure, or menopausal stage heterogeneity—may have influenced the outcomes. The reliance on self-reported instruments for symptom and sleep evaluation introduces possible recall or expectancy bias. Additionally, the 12-week duration and lack of post-intervention follow-up limit conclusions regarding long-term sustainability. Finally, environmental factors such as weather, terrain, and group dynamics could have influenced participant experience and adherence during the outdoor sessions.

Future research should employ randomized controlled designs to strengthen causal inference and directly compare barefoot walking with shod or other exercise modalities. Objective biomarkers—such as inflammatory cytokines, cortisol rhythms, heart rate variability, or blood viscosity—should be included to clarify the physiological mechanisms underlying observed improvements. Longitudinal studies are needed to evaluate whether continued barefoot walking sustains symptom relief and contributes to long-term physical and psychological health benefits in menopausal and postmenopausal women. In addition, qualitative studies exploring participants’ experiences and perceptions may provide valuable insights into motivational and adherence factors.

In addition, future investigations could compare the effects of walking on different surface types (e.g., concrete, grass, forest trails) and explore the feasibility of indoor barefoot walking programs during winter months. Furthermore, studies assessing potential risks such as foot injuries or infections would help establish safety guidelines for wider implementation.

Finally, integrating barefoot walking into community-based nursing and public health programs could represent an innovative, sustainable, and culturally adaptable approach to promoting menopausal well-being and healthy aging.

This study was designed to compare a structured barefoot walking program with a non-exercise control condition to determine whether barefoot walking offers benefits beyond usual daily activity in middle-aged women experiencing menopausal symptoms. As the primary goal was not to differentiate between barefoot and shod walking, the control group did not participate in structured walking. Future studies comparing barefoot and shod walking under identical exercise conditions are warranted to clarify whether the observed benefits are attributable to walking per se or specifically to walking barefoot. Therefore, the current findings should not be interpreted as evidence that barefoot walking is superior to shod walking; rather, they indicate that engaging in a structured barefoot walking program was beneficial compared with no structured physical activity.

## 5. Conclusions

This study demonstrated that a 12-week barefoot walking program significantly improved menopausal symptoms, sleep quality, stress, and quality of life in middle-aged women, while body composition remained unchanged. These findings suggest that barefoot walking is an effective, safe, and accessible nonpharmacological strategy to enhance psychosomatic well-being during the menopausal transition.

The observed benefits likely reflect the combined effects of moderate physical activity, natural environmental exposure, and direct foot-to-ground contact, which together promote relaxation and emotional balance. Given its simplicity and low cost, barefoot walking can be feasibly integrated into lifestyle-based health promotion and menopause management programs.

Future randomized controlled trials incorporating biological markers and long-term follow-up are needed to confirm the physiological mechanisms and sustainability of these effects. In summary, barefoot walking represents a practical and restorative intervention that supports both physical and psychological health in midlife women.

## Figures and Tables

**Table 1:** Barefoot walking program.

Exercise	Duration	Frequency	Distance	Activity Durations
Barefoot walking	12 weeks	3 times a week	2.7 km	Warm-up: 5 min Main exercise: 50 min Cool-down: 5 min

**Table 2:** Baseline characteristics and homogeneity of primary and secondary outcomes between groups (N = 60).

Characteristic	Exp. (*n* = 29)	Cont. (*n* = 31)	χ^2^ or t or z	*p*
	*n* (%)/M ± SD/Med (IQR)			
Age (yr)	56.86 ± 5.18	56.26 ± 4.50	0.48	0.631
Height (cm)	156.03 ± 6.87	157.26 ± 6.52	−0.71	0.482
Weight (kg)	57.22 ± 9.04	60.39 ± 7.99	−1.44	0.155
Religion			0.20	0.655
Yes No	21 (72.4) 8 (27.6)	24 (77.4) 7 (22.6)		
Education Level			1.75	0.185
≤High School ≥College	19 (65.5) 10 (34.5)	25 (80.6) 6 (19.4)		
Employment status			0.05	0.833
Yes No	24 (82.8) 5 (17.2)	25 (80.6) 6 (19.4)		
Marital status			<0.01 ^(a)^	>0.999
Married Others	27 (93.1) 2 (6.9)	29 (93.5) 2 (6.5)		
Primary outcomes				
MRS	19.00 (17.50)	16.00 (13.00)	−1.31	0.192
Sleep quality	41.28 ± 11.87	43.00 ± 13.44	−0.53	0.601
Stress	43.00 (60.50)	25.00 (47.00)	−1.27	0.206
QoL	74.00 (21.00)	74.00 (16.00)	−0.37	0.711
Secondary outcomes (body composition indices)				
BMI (kg/m^2^)	23.70 ± 2.42	25.09 ± 3.15	−1.90	0.062
SMM (kg)	21.08 ± 2.78	21.38 ± 2.41	−0.45	0.655
BFM (kg)	18.64 ± 5.58	22.93 ± 5.31	−3.05	0.003
FFM (kg)	39.03 ± 4.66	39.02 ± 4.36	0.01	0.992
TBW (L)	28.63 ± 3.40	28.60 ± 3.20	0.03	0.974
BMR (kcal)	1207.28 ± 98.53	1212.74 ± 94.25	−0.22	0.827

Note. Exp., Experimental group; Cont., Control group; M, Mean; SD, Standard deviation; Med, Median; IQR, Interquartile range; MRS, Menopause rating scale; QoL, Quality of life; BMI, Body mass index; SMM, Skeletal muscle mass; BFM, Body fat mass; FFM, Fat-free mass; TBW, Total body water; BMR, Basal metabolic rate. ^(a)^ By Fisher’s exact test.

**Table 3:** Effects of barefoot walking on primary outcomes: menopausal symptoms, stress, and quality of life (N = 60).

Variables	Pretest M ± SD	Posttest M ± SD	Med (IQR)	U	z ^(a)^	*p*	r [95% CI]
MRS							
Exp. (*n* = 29) Cont. (*n* = 31)	19.66 ± 8.78 16.68 ± 6.28	9.52 ± 5.28 17.48 ± 7.17	−11.0 (7.50) 0.0 (6.00)	73.00	−5.59	<0.001	–0.72 [–0.83, –0.57]
Stress							
Exp. (*n* = 29) Cont. (*n* = 31)	49.52 ± 36.20 36.94 ± 31.96	16.76 ± 16.66 36.19 ± 33.63	−24.0 (54.50) 0.0 (15.25)	207.50	−3.58	<0.001	–0.46 [–0.64, –0.24]
QoL							
Exp. (*n* = 29) Cont. (*n* = 31)	74.34 ± 13.41 76.84 ± 14.82	90.45 ± 13.15 80.10 ± 13.76	15.0 (25.00) 2.5 (10.75)	215.50	−3.47	0.001	–0.45 [–0.63, –0.22]

Note. Exp., Experimental group; Cont., Control group; M, Mean; SD, Standard deviation; Med, Median; IQR, Interquartile range; MRS, Menopause rating scale; QoL, Quality of life. ^(a)^ By Mann–Whitney U test; 95% confidence intervals were obtained via Fisher’s z transformation.

**Table 4:** Effects of barefoot walking on primary outcome: sleep quality (N = 60).

Variables	Pretest M ± SD	Posttest M ± SD	Source	F ^(a)^	*p*	Partial η^2^ [95% CI]
Sleep Quality						
Exp. (*n* = 29) Cont. (*n* = 31)	41.28 ± 11.87 43.00 ± 13.44	52.41 ± 15.23 45.00 ± 11.50	G T G*T	0.94 15.56 7.53	0.337 <0.001 0.008	0.016 [0.000, 0.136] 0.212 [0.055, 0.409] 0.115 [0.009, 0.298]

Note. Exp., Experimental group; Cont., Control group; M, Mean; SD, Standard deviation; G, Group; T, Time; G*T, Group*Time. ^(a)^ By repeated measures ANOVA

**Table 5:** Effects of barefoot walking on secondary outcomes: body composition indices (N = 60).

Variables	Pretest M ± SD	Posttest M ± SD	Source	F	*p*	Partial η^2^ [95% CI]
BMI (kg/m^2^) ^(a)^						
Exp. (*n* = 29) Cont. (*n* = 31)	23.70 ± 2.42 25.09 ± 3.15	23.68 ± 2.50 24.94 ± 3.27	G T G*T	3.22 1.38 0.64	0.078 0.244 0.427	0.053 [0.000, 0.209] 0.023 [0.000, 0.153] 0.011 [0.000, 0.122]
SMM (kg) ^(a)^						
Exp. (*n* = 29) Cont. (*n* = 31)	21.08 ± 2.78 21.38 ± 2.41	21.16 ± 3.12 21.57 ± 2.44	G T G*T	0.28 0.89 0.17	0.601 0.350 0.679	0.005 [0.000, 0.102] 0.015 [0.000, 0.133] 0.003 [0.000, 0.093]
BFM (kg) ^(b)^						
Exp. (*n* = 29) Cont. (*n* = 31)	18.65 ± 5.58 22.93 ± 5.31	18.44 ± 5.45 22.28 ± 5.50	G C G*C	1.01 901.98 0.61	0.321 <0.001 0.438	0.018 [0.000, 0.144] 0.942 [0.904, 0.965] 0.011 [0.000, 0.125]
FFM (kg) ^(a)^						
Exp. (*n* = 29) Cont. (*n* = 31)	39.00 ± 4.74 39.02 ± 4.36	39.15 ± 5.18 39.27 ± 4.44	G T G*T	<0.01 1.00 0.06	0.957 0.322 0.806	<0.001 [0.000, 0.075] 0.017 [0.000, 0.138] 0.001 [0.000, 0.080]
TBW (L) ^(a)^						
Exp. (*n* = 29) Cont. (*n* = 31)	28.65 ± 3.46 28.60 ± 3.20	29.20 ± 4.02 28.75 ± 3.28	G T G*T	0.08 2.49 0.85	0.766 0.120 0.362	0.001 [0.000, 0.080] 0.042 [0.000, 0.190] 0.015 [0.000, 0.133]
BMR (kcal) ^(a)^						
Exp. (*n* = 29) Cont. (*n* = 31)	1207.28 ± 980.53 1215.37 ± 940.70	1212.79 ± 110.21 1179.54 ± 240.57	G T G*T	0.15 0.49 0.91	0.699 0.487 0.343	0.003 [0.000, 0.093] 0.009 [0.000, 0.116] 0.016 [0.000, 0.136]

Note. Exp., Experimental group; Cont., Control group; M, Mean; SD, Standard deviation; BMI, Body mass index; SMM, Skeletal muscle mass; BFM, Body fat mass; FFM, fat-free mass; TBW, Total body water; BMR, Basal metabolic rate; G, Group; T, Time; G*T, Group* Time; C, Covariate; G*C, Group* Covariate. ^(a)^ By repeated measures ANOVA. ^(b)^ By ANCOVA.

## Data Availability

Please contact the corresponding author for data availability.
